# Osseous Genioplasty and Its Impact on Airway Volume—What is the Evidence?

**DOI:** 10.1093/asjof/ojaf059

**Published:** 2025-06-10

**Authors:** Martin Kauke-Navarro, Leonard Knoedler, Lars Stucki, Felix J Klimitz, Omar Allam, Samuel Knoedler, Miguel Carlo Navarro, Paula Flores-Pérez, Albert L Rancu, Max Heiland, Michael Alperovich, Ali-Farid Safi

## Abstract

Osseous genioplasty (OG) improves facial aesthetics by 3-dimensionally repositioning the chin and associated soft tissues. Although its effects on airway volumes and obstructive sleep apnea (OSA) have been investigated, the available evidence remains limited. The authors of this study performed a systematic review using the databases PubMed/MEDLINE, Web of Science, Google Scholar, Cochrane, and EMBASE through January 2025. Studies assessing the impact of OG on airway volumes and/or OSA metrics were included. Data on chin advancements, hyoid movements, and related outcomes were extracted. Nine studies met the inclusion criteria. Chin advancement ranged from 4 to 12.5 mm (mean: 7.3 mm), and hyoid advancement ranged from 2.17 to 10 mm (mean: 5.6 mm). Six studies assessed airway changes, with most reporting increases in airway size. Among those, 3 studies quantified volumetric changes. On average, total airway volume increased by 8.5%, with initial volumes averaging 17,956.5 mm^3^ and increasing to 19,467.5 mm^3^. The average absolute volume increase was 1511 mm^3^, with the greatest expansion observed in the oropharynx and hypopharynx. Other studies measured linear airway changes, with posterior airway space increasing by a mean of 2.9 mm. One study reported inconsistent results, with some cases demonstrating no significant airway changes postoperatively. These findings suggest a general trend of airway enlargement following intervention. Functional improvements included reductions in apnea–hypopnea index from 27.7 to 12.7 and from 12.4 to 4.4 in 2 studies, along with reported improvements in snoring severity. Genioplasty can enhance airway dimensions and may improve OSA symptoms through forward chin advancement and hyoid elevation. Although outcomes are consistently positive, standardized assessment methods are needed to optimize surgical techniques and evaluate long-term results.

**Level of Evidence**: 4 (Therapeutic)

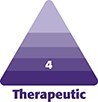

Obstructive sleep apnea (OSA) is one of the leading respiratory disorders, characterized by sudden episodes of apnea/hypopnea during sleep due to upper airway obstruction or narrowing.^1^ This condition can have profound adverse effects on quality of life and general health and is associated with metabolic disorders, such as obesity, Type 2 diabetes mellitus, hypertension, and cardiovascular disease.^2^ Surgical procedures for patients with OSA, such as maxillomandibular advancement (MMA), are well established in their ability to significantly open the airway and improve OSA-related metrics, including the apnea–hypopnea index (AHI), respiratory disturbance index, daytime sleepiness, snoring, and blood pressure.^3,4^ As a result, MMA is often considered the procedure of choice in patients with moderate-to-severe OSA. Alternative mandibuloplasty techniques, such as mandibular wing osteotomy, have also demonstrated efficacy in improving OSA symptoms, as measured by metrics like the Epworth Sleepiness Scale and AHI.^4^

Previous studies have emphasized the utility of advancement genioplasties in increasing the pharyngeal airway space by advancing the hyoid bone and tongue base through the genioglossus muscle attachments to the chin segment (genial tubercle).^5-8^ Similarly, a setback of the mandible/chin segment can lead to reduced upper airway volumes.^6,9^ The impact of isolated genioplasties on airway volumes and as an added benefit for patients undergoing cosmetic genioplasties has not been systematically evaluated.^9^ Although genioplasty cannot be presented as a primary procedure for OSA management in moderate-to-severe cases, examining its isolated effect on airway changes may provide valuable insights.

## METHODS

A systematic review was conducted by 2 independent reviewers (M.K.-N. and L.K.) to evaluate the impact of isolated genioplasty on airway volume. The systematic review was registered with the International Prospective Register of Systematic Reviews (PROSPERO identifier: CRD420251042447).

We strictly adhered to the principles of the Preferred Reporting Items for Systematic Reviews and Meta-Analyses guidelines (Supplemental Table).^10^ Based on insights from 3 sentinel articles, a comprehensive search string was developed to identify relevant studies.^6,7,9^ The search terms included: (“Genioplasty” OR “Chin Surgery” OR “Osseous Genioplasty” OR “Sliding Genioplasty” OR “Chin Advancement” OR “Chin Repositioning”) AND (“Airway” OR “Upper Airway” OR “Airway Volume” OR “Pharyngeal Airway” OR “Respiratory Changes”).

This search was applied across multiple databases (PubMed/MEDLINE, Web of Science, Google Scholar, Cochrane, and EMBASE) up to January 5, 2025. Filters were applied to include only human studies, full-text availability, and English-language publications. Additional screening criteria excluded studies that (1) examined genioplasty in conjunction with other procedures (eg, syndromic cases or concurrent orthodontic treatments) and (2) were technical reports lacking quantitative outcome measures or assessments. Further searches were performed using Google Scholar, focusing on cited and citing articles and cross-referenced sources. A search in the Cochrane Library was also conducted.

Eligible studies were limited to those analyzing isolated genioplasty and its effect on airway volume enhancement and/or OSA-related outcome metrics. Screening and data extraction were performed independently by both reviewers, with disagreements resolved by consulting a third reviewer (A.-F.S.).

## RESULTS

**Figure 1.**
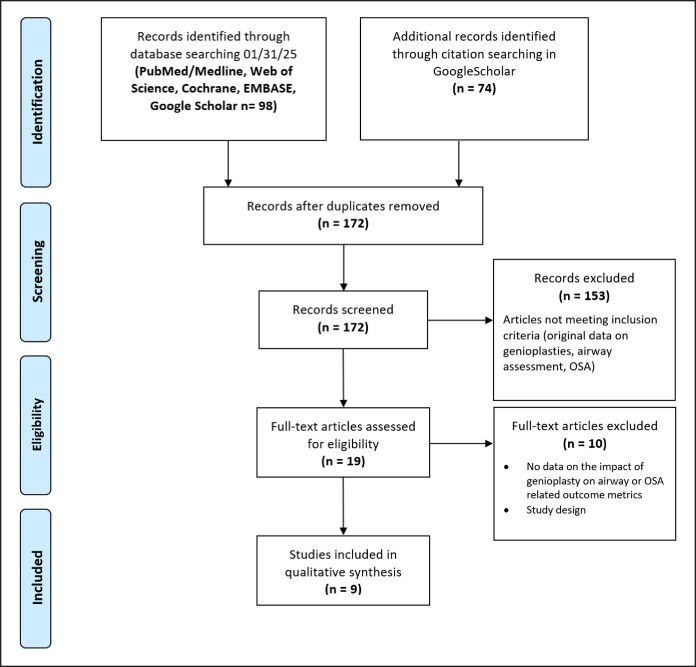
Preferred Reporting Items for Systematic Reviews and Meta-Analysis flow diagram of study identification, screening, and inclusion.

The initial database search yielded 98 studies. After applying filters for human studies, full-text availability, and English-language publications, 65 studies remained. Additional searches through citation tracking contributed 74 articles and 20 cross-referenced entries. After screening and eligibility assessment, a total of 9 studies were included, comprising 261 patients undergoing osseous genioplasty (OG; (Figure 1, Table 1).^6,7,9,11-16^ The studies were conducted across China, Spain, Japan, France, Brazil, the United States of America, and South Korea and included 2 prospective studies, 6 retrospective, and 1 case report. The mean age of patients ranged from 10 to 40 years. Gender distribution across studies totaled 150 males and 125 females, with some studies not reporting gender data.

**Table 1.** General Study Characteristics

Author	Year	Country	Study design	Sample size	Age (mean ± SD)	Gender (male:female)	Type of genioplasty/movement	Chin advancement (mm)	Hyoid advancement	Indication	Follow-up duration/schedule	Relevant complications
Du et al^6^	2017	China	Prospective	12	24.7 ± 4.8	18/17	Advancement, setback, combined	4-8 mm (mean 5.6 ± 2)	Not reported	OSA in obese patients	1 week postoperative, max 1 year follow-up	None reported
Valls-Ontañón et al^9^	2024	Spain	Retrospective	44	33.4 ± 8.4	13/31	Advancement (39 sagittal, 3 retrusion), vertical (5 upward, 3 downward), centering (6 cases)	Mean sagittal: 4.24 ± 1.96; vertical: 4.69	Vertical ascent: 1.78 ± 1.79 mm	Airway improvement, cosmetic reasons	6-12 months	None reported
Kino et al^7^	2023	Japan	Retrospective	6	16 (range, 10-23)	4/2	Sliding genioplasty; distraction osteogenesis in 1 case	10 mm (range, 7-13 mm)	Point B to hyoid: shortened ∼5 mm	Recurrent OSA after previous surgeries	6 months	None reported
Chen et al^11^	2020	China	Retrospective	14	23.9 ± 3.2	9/5	Sliding genioplasty with clockwise rotation	7.3 ± 1.7 (range, 4-10 mm); downward: 3.6 ± 1.3	Advancement: 0.64 mm; upward: 0.9 mm	Skeletal Class II with airway concerns (no OSA)	6 months	None reported
Bedoucha et al^12^	2015	France	Retrospective Comparative	23	12.9 (range, 10-14)	11/12	Sliding genioplasty with orthodontic treatment	Not reported	Forward: +2.17 mm; upward: +3.9 mm	Mouth breathing, hyperdivergent profile	Growth phase (∼4 years)	No major complications
dos Santos et al^13^	2007	Brazil	Prospective	10	Not reported	Not reported	Sliding genioplasty	8-10 mm	Not reported	Mild/moderate OSA with retrognathia	6 months	None significant (mild swelling)
Omori et al^14^	2024	Japan	Case report	1	10	1/0	Advancement with distraction osteogenesis	12.5 mm	10 mm advancement	Pediatric OSA with tongue base obstruction	2 years	None reported
Chan and Ducic^16^	2016	USA	Retrospective	126	39.8 ± 14.4	81/45	Sliding advancement genioplasty	Not reported	Not reported	OSA and cosmetic	Not reported	6.3% (2 plate extrusion, 2 infections, 3 mental nerve injury, 1 tooth root injury)
Choi et al^15^	2019	South Korea	Retrospective	25	25.3 ± 5.2	13/12	Hat-shaped osteotomy, sliding genioplasty	8.8 mm (range, 6-10 mm)	Not reported	Retrogenia and snoring	12 months	None reported

OSA, obstructive sleep apnea; SD, standard deviation.

Sliding genioplasty was the most frequently performed technique, with some studies reporting on distraction osteogenesis, hat-shaped osteotomy, and additional vertical and centering modifications. The degree of chin advancement varied, with reported mean values ranging from 4 to 12.5 mm. Hyoid advancement was reported in select studies, ranging from 0.64 to 10 mm. The primary indications for genioplasty included retrognathia, airway concerns (OSA or snoring), and cosmetic correction. Follow-up durations ranged from 6 months to 4 years, with most studies assessing outcomes within the first postoperative year. Complication rates were low, with most studies reporting no major complications. One study reported a 6.3% complication rate, including plate extrusion, infections, mental nerve injury, and tooth root injury.

Furthermore, we extracted data on functional benefits such as OSA improvement following genioplasty. Table 2 highlights the impact of genioplasty on OSA. Kino et al and dos Santos et al reported significant reductions in the AHI, with Kino et al observing a decrease from 27.7 to 12.7 and dos Santos et al reporting a drop from 12.4 to 4.4.^7,13^ Additionally, Omori et al documented a reduction in oxygen desaturation index (ODI) from 6.1 to 0.81 and an increase in the lowest SpO_2_ from 85% to 92%.^14^ Subjective snoring improvements were prominent in Choi et al, with Stanford scale scores decreasing from 8.68 to 4.08.^15^

**Table 2.** Obstructive Sleep Apnea Metrics Following Genioplasty

Author	AHI (mean ± SD)	Endpoints (ODI, SpO_2_, snoring)	Other
Du et al^6^	Not reported	Not reported	Focus on cephalometric changes; no OSA-specific metrics assessed
Valls-Ontañón et al^9^	Not reported	Not reported	Focus on volumetric airway improvements (UAV +9.89%, oropharynx +16.8%)
Kino et al^7^	Significant improvement: 27.7 ± 11.6 → 12.7 ± 3.4	Lowest SpO_2_: no significant change Average SpO_2_: no significant change	Used polysomnography; significant AHI improvement; no volumetric analysis
Chen et al^11^	Not reported	Not reported	Volumetric analysis only; significant hypopharynx and total PAS volume changes
Bedoucha et al^12^	Not reported	Not reported	Focus on linear airway measurements (eg, ELP, EVP); no functional OSA metrics included
dos Santos et al^13^	Significant improvement: 12.4 ± 4.6 → 4.4 ± 5.7	Not reported	Used polysomnography; PAS improvement from 7.9 to 10.8 mm (*P* < .001)
Omori et al^14^	Preoperative: 5.4 (no postoperative data)	ODI: significant improvement (6.1 → 0.81) Lowest SpO_2_: improved (85% → 92%)	Functional results include ODI and SpO_2_ improvement; no volumetric or linear airway analysis
Chan and Ducic^16^	Not reported	Not reported	92% of patients with OSA improved, 72% reduced CPAP dependency; no airway measurements
Choi et al^15^	Not reported	Subjective snoring: improved (8.68 → 4.08) Snoring improvement: 88% reported	88% reported snoring improvement; subjective snoring scale used; no airway measurements

AHI, apnea–hypopnea index; CPAP, continuous positive airway pressure; ELP, epiglottic position; EVP, epiglottic-vallecula position; ODI, oxygen desaturation index; OSA, obstructive sleep apnea; PAS, posterior airway space; SD, standard deviation; SpO_2_, peripheral capillary oxygen saturation; UAV, upper airway volume.

Airway change assessment was done using different radiological methods such as computed tomography (CT), cone-beam CT (CBCT), and lateral cephalograms. Linear and/or cross-sectional measurements were provided in 6 studies.^6,7,9,11-13^ Volumetric changes were assessed in 3 studies.^6,9,11^ Findings are summarized in Table 3. In brief, Du et al performed segmentation of the nasopharynx (palatopharyngeal, defined by a plane passing through the posterior nasal spine and sella, perpendicular to the sagittal plane), the oropharynx (defined as the airway between the soft palate plane and the most posterior point of the tongue base [PTA]) and hypopharynx (PTA to epiglottis plane [horizontal plane through the roof of the epiglottis]).^6^ The authors reported increases in total airway volume (+1493.9 mm^3^) and oropharyngeal volume (+218.5 mm^3^) following a mean chin advancement of 5.6 mm.

**Table 3.** Radiological Airway Evaluation Following Genioplasty

Author	Significant airway volume improvement documented	Chin advancement mean, SD (mm)	Radiological method for airway assessment	Type of measurement	Reference points	Significant changes (pre-post)	Improvement (difference)
Du et al^6^	Yes (linear, volumetric)	5.6 ± 2	CT	Linear, CSA, volume	H-N(A-P), H-N(L-R), H-N(T-B), SPA, PTA, EA; VP, VO, VG, VL	Linear (mm): H-N(A-P): 48.4 → 44.6 Area (mm^2^): SPA: 143.9 → 152.9; PTA: 145.6 → 158.2; EA: 203.3 → 224.6 Volume (mm^3^): VT: 20,919.8 → 22,413.7; VO: 6100.4 → 6318.9; VG: 4251.1 → 4833.2; VL: 3672.7 → 4247.5	Linear (mm): H-N(A-P): −3.8 Area (mm^2^): SPA: +9; PTA: +12.6; EA: +21.3 Volume (mm^3^): VT: +1493.9; VO: +218.5; VG: +582.1; VL: +574.8
Valls-Ontañón et al^9^	Yes (volumetric assessment)	Mean sagittal: 4.24 Mean vertical: 4.69	CBCT	UAV	Total airway, naso-, oro-, and hypopharynx	Total UAV: +9.89% (2817 ± 7256 mm^3^) Oropharynx: +16.8% (+2778.8 ± 6026 mm^3^) Chin centering: Median: Oropharynx: 8420 mm^3^, Total: 8651 mm^3^	Total UAV: +9.89% Oropharynx: +16.8%; Chin centering: +8420 mm^3^
Kino et al^7^	No (linear measurement)	10 (range, 7-13)	CT	Airway area (CSA)	Planes parallel to the orbito-meatal line at uvula, epiglottis, and C2–C7/Th1	No significant differences	Not provided
Chen et al^11^	Yes (volumetric assessment)	Avg: 7.3 (range, 4-10) Drop: 3.6 (range, 2-6)	CBCT, lateral cephalogram (LC)	LC (width), CBCT (CSA, volume)	LGo, LH, *S*_mean_, *S*_min_, *S*_tongue_, *S*_hyoid_; CV1-CV4 planes	Width (mm): LGo: 11.15 → 12.05; LH: 12.64 → 14.03 CSA (mm^2^): *S*_mean_: 269.9 → 298.7; *S*_min_: 150.7 → 203.7 Volume (mm^3^): total: 14,994 → 16,522; hypopharynx: 6269 → 7430	Width (mm): LGo: +0.9; LH: +1.38 CSA (mm^2^): *S*_mean_: +28.8; *S*_min_: +53.1 Volume (mm^3^): total: +1528; hypopharynx: +1161
Bedoucha et al^12^	Yes (linear measurement)	SNP angle: +3.16°	Lateral cephalogram	Cephalometric analysis (EVP, ELP, EHP)	EVP: posterior soft palate to pharyngeal wall; ELP: posterior tongue to pharyngeal wall	Linear (mm): EVP: +1.6 (95% CI, 0.7-2.5); ELP: +1.87 (95% CI, 0.33-3.4)	Linear (mm): EVP: +1.6; ELP: +1.87
dos Santos et al^13^	Yes (linear measurement)	9 mm	Lateral cephalogram	Linear measurements (PAS)	PAS: distance between tongue base and posterior pharyngeal wall	PAS (mm): 7.9 → 10.8	Linear (mm): PAS: +2.9
Omori et al^14^	Yes (linear measurement)	12.5 mm	Sleep MRI, LC	Linear measurements, drug-induced sleep endoscopy	Airway space behind the hyoid	Hyoid (mm): 3.75 → 7.55	Linear (mm): +3.8
Chan and Ducic^16^	Not evaluated	Not reported	Not reported	Not reported	Not reported	Not evaluated	Not evaluated
Choi et al^15^	Not evaluated	8.8 (range, 6-10)	Not reported	Not reported	Not reported	Not evaluated	Not evaluated

CBCT, cone-beam computed tomography; CSA, cross-sectional area; EA, epiglottis plane area; EHP, minimum hyopharyngeal dimension; ELP, minimum linguopharyngeal dimension; EVP, minimum velopharyngeal dimension; FH plane, Frankfort horizontal plane; H-N(A-P/L-R/T-B), hyoid-to-nasion distance (anterior-posterior/left-right/top-bottom); LC, lateral cephalogram; LGo, lateral distance to gonion; PAS, posterior airway space; PTA, posterior tongue plane area; SNPog, sella-nasion-pogonion angle; SPA, soft palate plane area; UAV, upper airway volume; VG, volume of glossopharynx; VL, volume of larynx; VO, volume of oropharynx; VT, total airway volume.

Valls-Ontañón et al utilized CBCT to measure total airway volume and its subdivisions without providing details on the boundaries of each airway subdivision.^9^ They reported a significant increase in oropharyngeal volume (+16.8%, *P* = .004), whereas nasopharyngeal volume changes were not statistically significant (+6.81%, *P* = .097), and hypopharyngeal volume showed no significant change (−7.56%, *P* = .210).

Chen et al utilized cervical vertebral landmarks (CV1-CV4) to define airway regions and assessed both cross-sectional area and total volume.^11^ They defined the nasopharynx as extending from the CV1 plane, parallel to the Frankfort horizontal plane, the oropharynx as the region between CV1 and CV2 planes, and the hypopharynx as spanning CV2 to CV4 planes. Their study found a significant increase in hypopharyngeal volume postgenioplasty (*P* < .05).

As summarized in Table 4, the researchers of most of the studies did not focus on metabolic outcomes. However, Du et al and dos Santos et al reported minor changes in BMI, with postoperative increases of +0.2 and +0.96, respectively.^6,13^ Rapid eye movement sleep duration improved significantly in dos Santos et al, increasing from 15.63 ± 8.66 to 20.29 ± 8.43 min.

**Table 4.** Metabolic Metrics Following Genioplasty

Author	BMI mean ± SD (kg/m^2^)	Metabolic outcomes (diabetes, cardiovascular, hypertension, REM sleep)
Du et al^6^	Preoperative: 25.6 postoperative (1 year): 25.8	None reported
Valls-Ontañón et al^9^	Not reported	None reported
Kino et al^7^	Not reported	None reported
Chen et al^11^	Not reported	None reported
Bedoucha et al^12^	Not reported	None reported
dos Santos et al^13^	Preoperative: 25.36 ± 2.98 Postoperative: 26.32 ± 3.23	Mean REM duration: 15.63 ± 8.66 → 20.29 ± 8.43
Omori et al^14^	Not reported	None reported
Chan and Ducic^16^	Not reported	None reported
Choi et al^15^	Postoperative: 21.28 (no preoperative data)	All patients reported positive influence on daily activities

REM, rapid eye movement; SD, standard deviation.

## DISCUSSION

Genioplasty is often performed for aesthetic enhancement of chin projection and contour, similar to the use of alloplastic chin implants.^17-19^ In addition to aesthetic benefits, OG may offer the added advantage of improving airway patency. Unlike other orthognathic procedures, the impact of isolated genioplasty on airway dimensions is less well established. In contrast, the positive effect of orthognathic movements such as bimaxillary advancements on airway volume is well documented and considered standard of care in the treatment of tongue-based airway obstructions.^20^ MMA leads to significant airway volume increases, whereas setbacks lead to decreased airway volume.^21^ In a retrospective cohort study of 103 patients undergoing different forms of orthognathic surgery (bimaxillary or isolated maxillary/mandibular advancement), patients undergoing bimaxillary advancement showed a total pharyngeal airway volume gain at the 1-year follow-up of 41.9%, outperforming monomaxillary (26%) and mandibular advancements (25%).^22^

### The Impact of Isolated Genioplasty on Upper Airway Volume

The impact of isolated genioplasty on airway volumes is less well understood. In this systematic review, we identified several studies that document positive airway changes following isolated genioplasty.^6,9,11-14^ Only 3 studies provide volumetric measurements of the upper airway based on 3D imaging modalities. Valls-Ontañón et al utilized image segmentation for volumetric airway analysis and reported an ∼10% increase in upper airway volume or 2817 mm^3^, following a mean sagittal chin advancement of 5.6 mm.^9^ The reported changes were predominately seen in the oropharynx (+16.8% volume increase, significant) and nasopharynx (+6.81%, not significant), with a relative reduction in hypopharyngeal volume (−7.56%). In another study by Du et al, a total airway volumetric gain of 1493 mm^3^ was recorded in patients who underwent a mean advancement of 5.6 mm.^6^ The majority of total volumetric change was attributed to the oropharynx and hypopharynx (both accounting for ∼85% of the airway change).^9^ Chen et al reported a total volumetric gain of 1528 mm^3^, with the majority of change seen in the hypopharynx (+1161 mm^3^). Linear measurements were utilized in 4 studies and corroborate positive changes in airway dimensions following genioplasty. Du et al reported a reduction in the hyoid-to-nasion (A-P) distance by −3.8 mm, indicating a shift in hyoid position after genioplasty.^6^ Bedoucha et al showed increased airway space in the velopharyngeal (+1.6 mm) and linguopharyngeal (+1.87 mm) planes.^12^ A posterior airway space increase of 2.9 mm was observed by dos Santos et al.^13^ Lastly, Omori et al provided a case report on 1 patient and found a 3.8 mm increase in retro-hyoidal airway space after a 12.5 mm chin advancement using distraction osteogenesis.^14^

One anatomically important region of the mandible in terms of airway collapse is the genial tubercle, the attachment site of the genioglossus muscle.^23^ Loss of muscle tone in this region is associated with OSA and snoring.^22^ Attachment of the genioglossus muscle to this region has led to consideration of the technique of genioglossus advancement for patients with mild-to-moderate OSA.^23,24^ To maximize the potential airway benefits of genioplasty, it is critical to ensure that the mandibular attachment of the genioglossus muscle, specifically the lingual surface of the symphysis menti, is included with the osteotomized segment.^15^ Forward repositioning of the osteotomized segment carrying the genioglossus attachment translates into increased tension at the base of the tongue, reducing airway collapse and enhancing airflow dynamics. Hence, variations in surgical technique—including whether this anatomical region is included—as well as differences in the magnitude of advancement, may influence the degree to which genioplasty affects airway volume. Preoperative planning using virtual surgical planning based on CT or CBCT can aid in accurately identifying the genial tubercle. In clinical practice, the use of cutting guides further facilitates the preservation of this key anatomical structure during osteotomy.

### Functional Outcomes: Obstructive Sleep Apnea and Symptom Improvement

Furthermore, we reviewed the articles for data on functional improvements after isolated genioplasty to quantify the clinical relevance of the findings. In the literature, patients with mandibular insufficiency who undergo mono- or bimaxillary advancement have well-documented benefits in terms of functional OSA metrics such as the AHI.^25,26^ The impact of isolated genioplasty is less well understood. Four studies reported on sleep-related functional improvements, although metrics varied, and outcome reporting was inconsistent between studies (Table 2). However, several studies suggested clinical benefits of isolated genioplasty. For example, both Kino et al and dos Santos et al reported significant reductions in the AHI following surgery, whereas Omori et al observed notable improvement in the ODI and lowest SpO_2_ levels.^7,13,14^ However, neither Omori et al nor Kino et al assessed volumetric airway changes. Choi et al reported significant subjective snoring improvement as reflected in the Stanford Snoring Scale, whereas Chan and Ducic noted that 92% of their patients experienced subjective OSA symptom relief, with 72% reducing continuous positive airway pressure dependency.^15,16^ In other studies that were included here, the authors did not provide data on OSA-related outcome metrics to assess the clinical relevance of the reported upper airway changes.

### Limitations

Airway assessment methods varied considerably, with inconsistent use of CT imaging and volumetric analysis. Lateral cephalograms and linear measurements, although employed in some studies, are inherently limited in their ability to evaluate changes in a 3-dimensional (3D) structure. Moreover, the limited application of image segmentation for volumetric assessment reduces the reliability of the findings presented herein.^27-29^ Differences in reported airway changes across studies may be attributed to several factors, including heterogeneity in imaging protocols, segmentation methods, and the anatomical landmarks used to define airway subregions. Additional variability may result from differences in patient positioning during imaging (both preoperatively and postoperatively), timing of postoperative scans, and surgical technique variations. Finally, differences in software algorithms for image segmentation likely contribute to variability in volumetric outcomes, particularly in subsite-specific areas, such as the hypopharynx. This variability highlights the need for standardized radiological assessments and additional studies that use image segmentation based on 3D scans.

Lastly, follow-up periods were generally short and varied, limiting the ability to assess the long-term effects of genioplasty on airway volume and function. Functional outcome parameters were reported in only 4 studies, further restricting the generalizability of the results. In addition, the small number of included studies and patients, along with missing or incomplete data in the primary literature, further limits the strength of the conclusions that can be drawn.

## CONCLUSIONS

Isolated genioplasty can enhance airway dimensions and may improve OSA symptoms through chin advancement and hyoid elevation. The current literature lacks sufficient long-term data to determine the factors influencing airway modifications postgenioplasty and how this may impact OSA-related metrics, highlighting the need for further well-designed studies.

## Supplementary Material

ojaf059_Supplementary_Data
